# A harmonized global nighttime light dataset 1992–2018

**DOI:** 10.1038/s41597-020-0510-y

**Published:** 2020-06-04

**Authors:** Xuecao Li, Yuyu Zhou, Min Zhao, Xia Zhao

**Affiliations:** Department of Geological and Atmospheric Sciences, Iowa State University, Ames, IA 50011 USA

**Keywords:** Environmental impact, Sustainability

## Abstract

Nighttime light (NTL) data from the Defense Meteorological Satellite Program (DMSP)/Operational Linescan System (OLS) and the Visible Infrared Imaging Radiometer Suite (VIIRS) on the Suomi National Polar-orbiting Partnership satellite provide a great opportunity for monitoring human activities from regional to global scales. Despite the valuable records of nightscape from DMSP (1992–2013) and VIIRS (2012–2018), the potential of the historical archive of NTL observations has not been fully explored because of the severe inconsistency between DMSP and VIIRS. In this study, we generated an integrated and consistent NTL dataset at the global scale by harmonizing the inter-calibrated NTL observations from the DMSP data and the simulated DMSP-like NTL observations from the VIIRS data. The generated global DMSP NTL time-series data (1992–2018) show consistent temporal trends. This temporally extended DMSP NTL dataset provides valuable support for various studies related to human activities such as electricity consumption and urban extent dynamics.

## Abstract (summary)

**Table:** 

Measurement(s)	nighttime light • light
Technology Type(s)	satellite imaging • Visible Infrared Imaging Radiometer Suite
Factor Type(s)	year of data collection • spatial resolution
Sample Characteristic - Environment	city
Sample Characteristic - Location	Earth (planet)

Machine-accessible metadata file describing the reported data: 10.6084/m9.figshare.12312125

## Background & Summary

The nighttime light (NTL) data from the Defense Meteorological Satellite Program (DMSP) - Operational Linescan System (OLS) are a primary data source to measure human activities from local, regional to global scales^1–4^. Compared to other remote sensing satellite observations, the NTL data can quantitatively characterize the intensity of the socioeconomic activities and urbanization^5^. Because of the global coverage and long temporal span, the DMSP NTL data have been extensively used in studies such as electricity consumption^6–9^, socioeconomic activities^10,11^, light pollution^12–15^, urban ecosystems^16^, and urban extent mapping^17,18^. However, the DMSP NTL data are not available to public after 2013, which notably limits the use of DMSP NTL data in urban studies.

The advent of the new generation of global NTL observations from the Visible Infrared Imaging Radiometer Suite (VIIRS) instrument makes it possible to continue the global NTL monitoring after 2012. Compared with the DMSP NTL data, both the spatial and radiometric resolutions of the VIIRS data have been notably improved, leading to fewer over-glow effects and spatially more explicit lights within the city^19^. Records in the VIIRS data are the detected radiance of city lights around the spectrum of 505–890 nm^5^. Although the new generation of the VIIRS NTL data are advanced in their capacities of monitoring city lights at night, the available temporal span is only available from 2012 to present, resulting in a relatively short period for exploring dynamics of human activities. Hence, a harmonization of NTL observations from DMSP and VIIRS data is highly needed^20,21^.

Although several studies have been performed to integrate NTL observations from the DMSP and VIIRS data, most of them focus on local scales or specific datasets. Li, *et al*.^22^ estimated the dynamics of city light in Syria’s major cities from 2011 to 2017 through converting the monthly VIIRS data into DMSP-like data. Zheng, *et al*.^23^ converted the VIIRS data to the DMSP-like radiance data in China through 1996 to 2017 using a geographically weighted regression approach. Essentially, this model was developed based on temporally extrapolated DMSP radiance data in 2012, and it contains uncertainties when directly applying this model in following years. Zhao, *et al*.^24^ integrated the DMSP and VIIRS NTL data using a sigmoid function model in Southeast Asia from 1992 to 2018. Parameters in this approach require additional calibration for global-scale application. Overall, these studies of integrating the NTL data from DMSP and VIIRS are limited (1) at local or regional scales without a global perspective^22–25^ and (2) using specific radiance data rather than the stable DMSP NTL dataset^23^. In addition, the consistency of DMSP NTL time series data across years (i.e., 1992–2013) is the premise of integrating these two data sources^26^, and this is not well addressed in most previous studies, particularly at the global scale.

There are two main difficulties in generating a temporally consistent and up-to-date DMSP NTL time series dataset at the global scale, by integrating NTL observations from DMSP and VIIRS. First, the stable DMSP NTL data should be inter-calibrated globally from 1992 to 2013. Although studies about the inter-calibration of DMSP NTL data have been carried out widely, most of them focused on local scales and the derived results are sensitive to the selected reference region and year^27–31^. Second, an efficient and robust approach should be developed to convert the VIIRS radiance data to DMSP-like NTL data. Existing approaches for this issue are limited in their robustness when extending them to other regions^22,24^ and also depend on other ancillary datasets (e.g., survey socioeconomic data and specific NTL radiance data)^23,25^. Few attempts have been made at the global scale, although such a long span and consistent DMSP-like time series dataset is highly needed in a variety of global urban studies.

## Methods

### Data collection

NTL observations from DMSP and VIIRS data are our main data sources in this study. First, we downloaded the stable DMSP/OLS NTL (version 4) from the Payne Institute for Public Policy under the Colorado School of Mines (https://eogdata.mines.edu/dmsp/downloadV4composites.html). Records in the DMSP NTL data are the digital number (DN) values, ranging from 0 to 63. The spatial resolution of DMSP NTL data is 30 arc-seconds, with a near-global coverage of 180°W to 180°E in longitude and 65°S to 75°N in latitude^32^. The temporal span of DMSP NTL data is from 1992 to 2013, with observations from different satellites (e.g., F10, F12, F14, F15, F16, and F18). Given that the DMSP NTL time series data are not comparable across years due to the lack of on-board calibration, varied atmospheric conditions, satellite shift, and sensor degradation^24,32^, we improved the raw DMSP NTL data using a stepwise calibration approach and generated a temporally consistent NTL dataset^26^. The derived DMSP NTL time series data outperform those using traditional approaches^28,33^ regarding the temporal trend of global total DNs from different satellites and years, as well as the correlation with the electricity consumption data^26^.

Second, we collected the version 1 suite of global average radiance composite images from the VIIRS Day/Night Band (DNB) data (https://eogdata.mines.edu/download_dnb_composites.html). Different from the DN values in the DMSP data, the records in the VIIRS are the radiance of NTL data with an improved radiometric resolution. The spatial resolution of VIIRS product is 15 arc-seconds, across the latitudinal zone of 65°S-75°N^5^. The data version used in our study is the monthly “VIIRS Cloud Mask (vcm)” from 2012 to 2018, which excludes observations affected by stray light^24^. Also, effects caused by biogeophysical processes such as seasonal dynamics of vegetation and snow have been corrected in the monthly composite of VIIRS NTL data using the bidirectional reflectance distribution function^34^. The monthly VIIRS NTL data were further preprocessed and composited as annual time series data.

### Framework

**Fig. 1:**
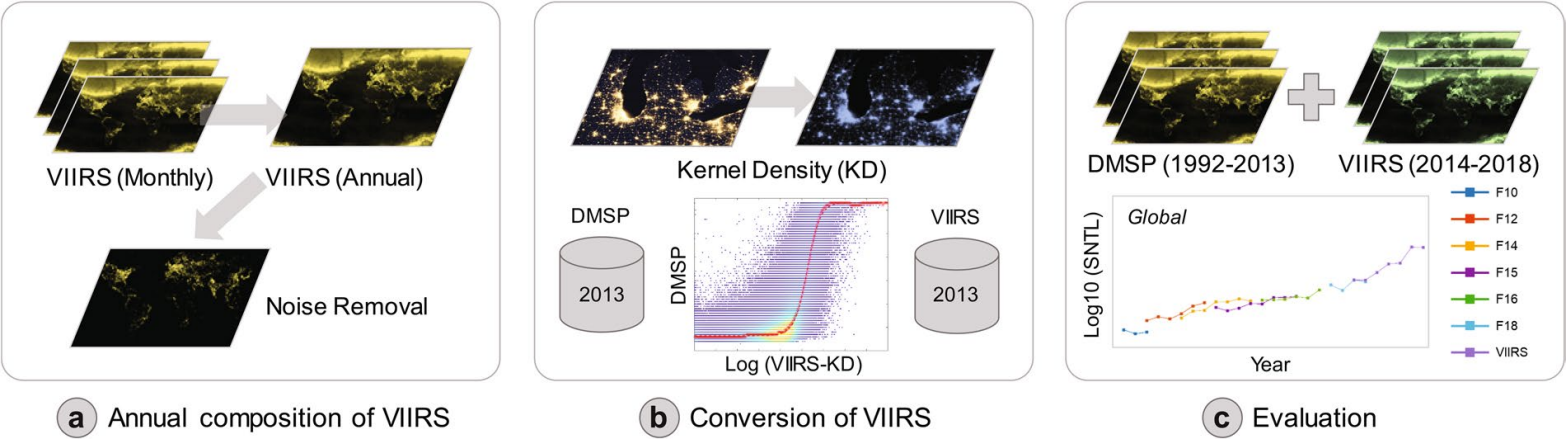
The proposed framework of generating a consistent global NTL time series data through integration of DMSP and VIIRS. Annual composition of VIIRS data (**a**); conversion of VIIRS data (**b**); and evaluation of generated global NTL time series data (**c**).

We generated the global harmonized NTL data from 1992 to 2018 through the integration of the DMSP and VIIRS data using the framework in Fig. 1. The proposed framework contains three major steps. First, we composited the annual VIIRS NTL radiance data from the monthly observations (Fig. 1a). Noises from aurora, fires, boasts, and other temporal lights were excluded during this step. Second, we quantified the relationship between processed VIIRS data and DMSP NTL data in 2013 using a sigmoid function (Fig. 1b). The processed VIIRS data have the same spatial resolution and similar radiometric characteristics as DMSP data. Third, we applied the derived relationship at the global scale to obtain the DMSP-like data from VIIRS, and finally generated the consistent NTL data by integrating the temporally calibrated DMSP NTL data (1992–2013)^26^ and DMSP-like NTL data from VIIRS (2014–2018) (Fig. 1c). The performance of generated global consistent NTL data was finally evaluated. Details of these steps were presented in following sections.

#### Annual composition of VIIRS data

**Fig. 2:**
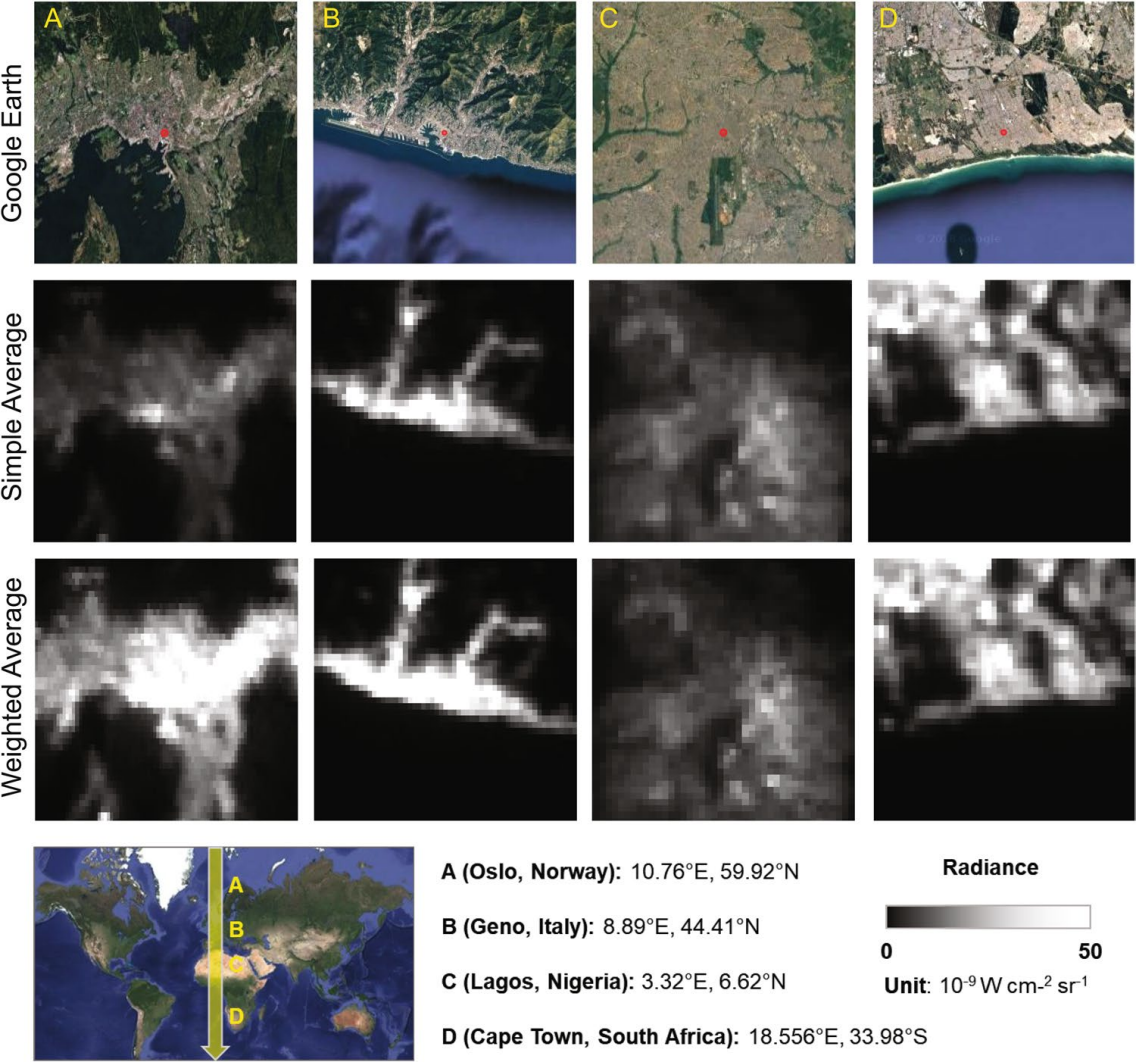
Comparison of the annual composition of VIIRS NTL radiance data from the monthly observations using simple average approach and weighted average approach (i.e., cloud-free observations). The spatial extent of each snapshot is 20 km × 20 km.

The global annual VIIRS NTL radiance data were composited from monthly observations after noise removal. First, due to cloud and solar illumination^5,34^, the quality of the monthly VIIRS radiance data is different across regions. In this study, we used the information of cloud-free coverage in each month as a weighting factor to generate the annual composition as follows:

1 $${W}_{cf}^{m}=c{f}_{m}\,/\,\mathop{\sum }\limits_{m=1}^{12}c{f}_{m}$$

2 $$VIIR{S}_{a}=\mathop{\sum }\limits_{m=1}^{12}VIIR{S}_{m}\ast {W}_{cf}^{m}$$

where ${W}_{cf}^{m}$ is the weight of cloud-free observation in month *m*, and $VIIR{S}_{a}$ is the annually composited radiance value from monthly data. *cf*_*m*_ and $VIIR{S}_{m}$ are the number of cloud-free observations and the radiance recorded in month *m*. The weighting average approach outperforms the simple average approach (i.e., by averaging the pixel brightness of all data in each year) because the quality (i.e., cloud coverage) of monthly VIIRS radiance data was considered in this approach. The resulting composited VIIRS radiance data can improve the data quality in urban domains, particularly for cities in middle- and high-latitude areas (Fig. 2). For example, the composite annual radiance in Olso (Norway) was notably improved using only cloud-free observations.

Second, we removed noises caused by aurora and temporal lights using a threshold approach. For regions in high latitudes, aurora is a widely seen phenomenon due to the solar wind, and it is a relatively strong disturbance for the detected NTL by sensors^34^. Regions with latitudes above 45°N or below 45°S were regarded as zones influenced by the aurora^34^. In this zone, we selected pixels where DMSP values in two temporally overlaid years (i.e., 2012 and 2013) are zeros (background) and the radiances in VIIRS are above zero. Lights of these pixels are likely attributed from the effect of aurora. We measured the mean ($\mu$) and standard derivation ($\sigma$) of VIIRS radiances, and excluded noises caused by aurora using the determined threshold of 1.5 ($\mu +3\sigma$), i.e., radiance above 1.5 × 10^−9^ W cm^−2^ sr^−1^ was regarded as lights from human activities. It should be noted that high latitude cities affected by aurora are not specifically corrected due to mixed signals of city lights and aurora, however, the proportion of them is very small (i.e., about 0.3%) (Fig. S1)^17^. For other regions, we used the empirical threshold of 0.3 to remove dim light noises caused by temporal lights from fires and boats^22,24,35^. In addition, using the boundary of potential urban cluster^17^, we excluded bright VIIRS pixels that are not relevant to human activities using the maximum radiance of NTL in urban domains. However, it is worth noting that some light sources such as lighted greenhouses are still included. Finally, we generated annual compositions of VIIRS data from 2012 to 2018 with notably reduced effects from noises and cloud. It is worth noting that the local overpass time of VIIRS data is around 1:30 am, which is different from the DMSP in about 9:00 pm^36^. The difference caused by overpass time is a potential factor influencing the conversion from VIIRS to DMSP-like data. Although our annual composition scheme of VIIRS data cannot exclude all noises caused by varying lighting sources, these uncertainties will be mitigated notably through spatial aggregation of VIIRS to DMSP using the kernel density approach and the proposed sigmoid function for conversion.

#### Conversion of VIIRS data

We converted annual VIIRS observations to the DMSP-like NTL data using a sigmoid function. First, we spatially aggregated the annual VIIRS radiance data (15 arc-seconds) to the same resolution as DMSP NTL data (30 arc-seconds) using the kernel density (KD) approach^22,24^. Considering the blurring effect of DMSP NTL data, a symmetric Gaussian point-spread function was used when implementing the aggregation algorithm. We used a moving circle window of 5 times of the VIIRS pixel size to implement the KD approach. Both the nighttime light brightness and the distance of surrounding lit pixels to the central point were considered in the derived kernel density map of VIIRS data. More details about this step can be found in Zhao, *et al*.^24^.

Second, we implemented a logarithmic transformation on derived kernel density maps from the VIIRS images. The radiance variation of aggregated VIIRS data from kernel density results is different across urban, suburban, and rural areas, showing a difference compared to the DMSP data. To address this problem, we performed a logarithmic transformation for the aggregated VIIRS data^37^ as Eq. (3).

3 $$Lo{g}_{{V}_{kd}}{\rm{=}}ln({V}_{kd}+1)$$

where $Lo{g}_{{V}_{kd}}$ is the logarithmic transformation of the aggregated kernel density result of VIIRS data ${V}_{kd}$. The number of one was added in Eq. (3) to avoid the invalid values generated during the logarithmic transformation. The magnitude differences between the high and low ${V}_{kd}$ were considerably reduced after this transformation.

**Fig. 3:**
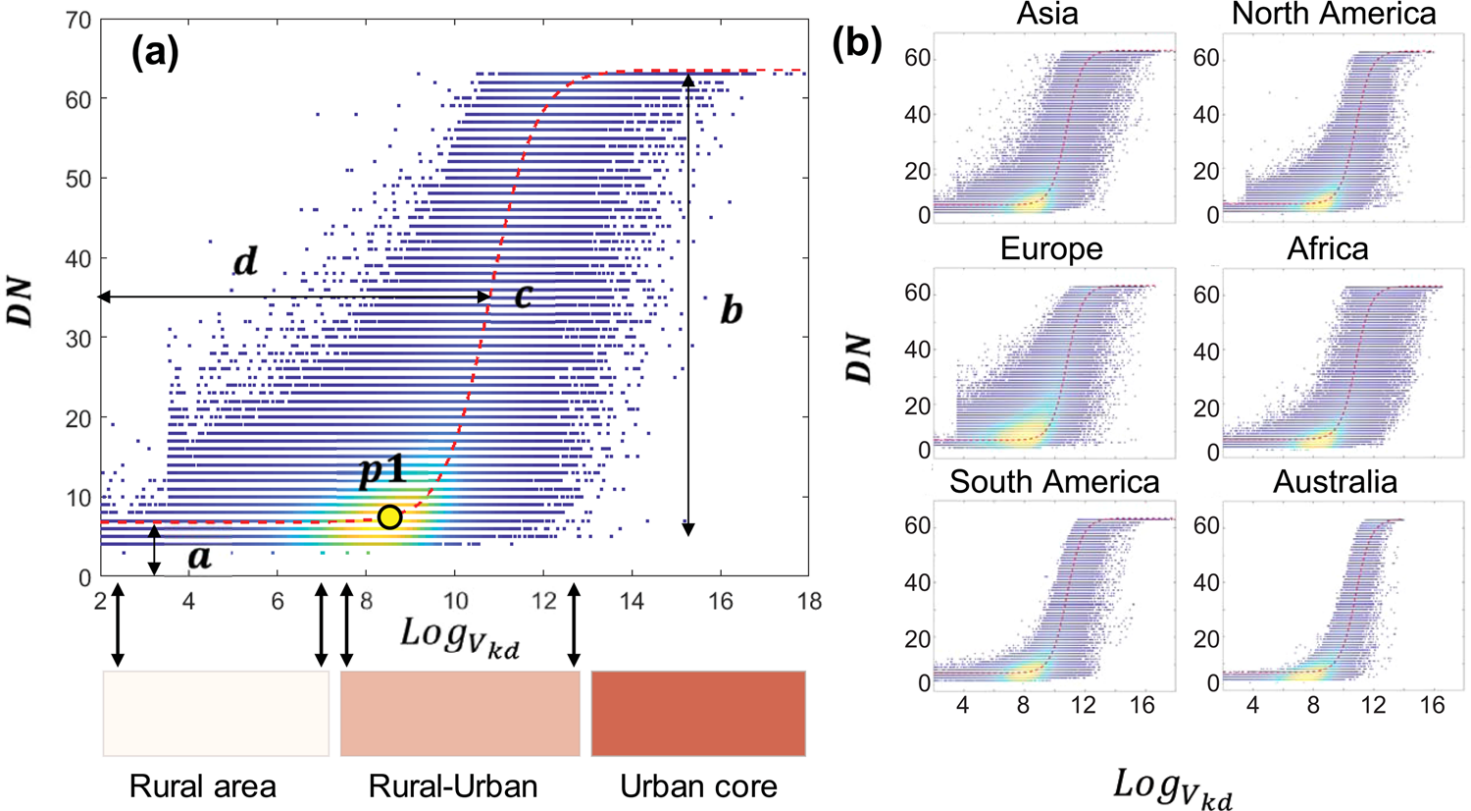
Illustration of the proposed sigmoid function to convert VIIRS radiance data to DMSP-like DN values at the global (**a**) and continental scales (**b**).

Third, we converted the $Lo{g}_{{V}_{kd}}$ from the VIIRS observations to DMSP-like DN values using a sigmoid function (Eq. 4 and Fig. 3) initially proposed by Zhao, *et al*.^24^ in Southeast Asia. As illustrated in Fig. 3a, the sigmoid function captures well the relationship between the DMSP DN and $Lo{g}_{{V}_{kd}}$, which correspond to different lit environments among regions of rural, rural-urban transition zones, and urban core. That is, the DN and $Lo{g}_{{V}_{kd}}$ are both lower in rural areas. When the $Lo{g}_{{V}_{kd}}$ falls within the urban-rural transition zones, the DN value rapidly increases along with the increase of $Lo{g}_{{V}_{kd}}$. For those lit pixels close to the urban core, where DNs in DMSP are saturated^1^, the increase of DN along with the $Lo{g}_{{V}_{kd}}$ is almost plateaued. These three different relationships can be captured using the sigmoid function with four parameters (i.e., *a*, *b*, *c* and *d*). We used year 2013 in developing the relationship because the VIIRS data in 2012 is only available from April. The derived sigmoid model at the global level is robust when applying it at the continental scales (Fig. 3b) and in example countries (Fig. S2), using the determined parameters of *a*, *b*, *c* and *d* as 6.5, 57.4, −1.9, and 10.8, respectively.

4 $$f\left(x\right)=a+b\left(\frac{1}{1+{e}^{-c(x-d)}}\right)$$

where $f\left(x\right)$ is the DMSP-like DN value using the logarithmic function from the aggregated kernel density value of VIIRS *x* (i.e., $Lo{g}_{{V}_{kd}}$). *a*, *b*, *c* and *d* are parameters in the logarithmic function.

#### Evaluation

We evaluated the integrated DMSP NTL time series data spanning from 1992 to 2018. First, we compared histograms of the observed DMSP DNs and simulated DMSP-like NTL data in two overlaid years of 2012 and 2013. Second, we compared the temporal pattern (1992–2018) of the harmonized NTL dataset using indicators of the sum of NTL (SNTL) DN values and the number of lit pixels, at the global scale. Third, we compared spatial patterns of observed and simulated DNs in metropolitan areas in the US and China.

## Data Records

The harmonized global NTL time series data include the stepwise calibrated stable DMSP NTL observations from 1992 to 2013, i.e., F10 (1992–1994), F12 (1995–1996), F14 (1997–2003), F16 (2004–2009), and F18 (2010–2013), and the simulated DMSP-like DNs from the VIIRS radiance data (2014–2018). The spatial resolution of this dataset is 30 arc-seconds. The uploaded data at the figshare repository (https://doi.org/10.6084/m9.figshare.9828827.v2)^38^ were tagged in GEOTIFF file format. These data can be processed by GIS software such as QGIS.

## Technical Validation

### Histograms of observed and simulated DN values

**Fig. 4:**
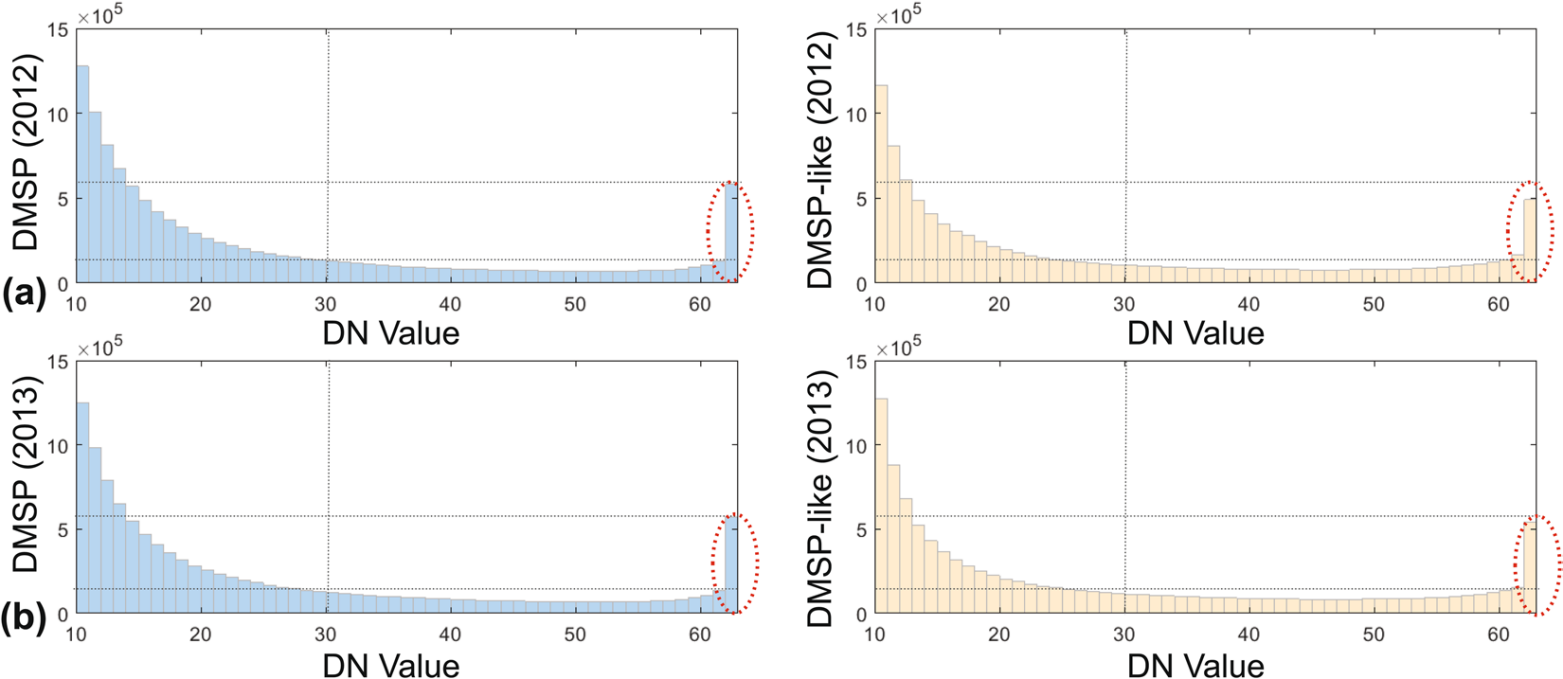
Comparison of histograms of NTL data from DMSP and VIIRS data in 2012 (**a**) and 2013 (**b**) at the global scale. Red ellipses represent DN pixels larger than 60.

At the global scale, the DN distribution of simulated DMSP-like NTL from VIIRS data agrees well with that from the observed NTL from the inter-calibrated DMSP data (Fig. 4). We selected years of 2012 and 2013 for comparison with the overlapped DMSP and VIIRS data. Given that lit areas representing certain human activities always grow slightly around their light source due to the blooming effects, most of the DMSP NTL data with low DN values on the periphery of the highly-lighted areas are not counted as urban areas^39^. Therefore, the non-lit areas with low DN values (DN < = 10) were removed in our analysis^17^. Overall, the derived histograms between the DMSP and DMSP-like data are similar. However, due to the saturation effect of DMSP NTL data, the number of pixels with DN values larger than 60 increases notably (red ellipses in Fig. 4), which is mainly from the plateaued DN values in the sigmoid function. In addition, the number of simulated DN pixels in 2012 was slightly lower compared to 2013 (red ellipses in Fig. 4). This is mainly attributed to the fact that the sigmoid function was calibrated using the observations in 2013.

### Temporal trend of NTL time series from 1992 to 2018

**Fig. 5:**
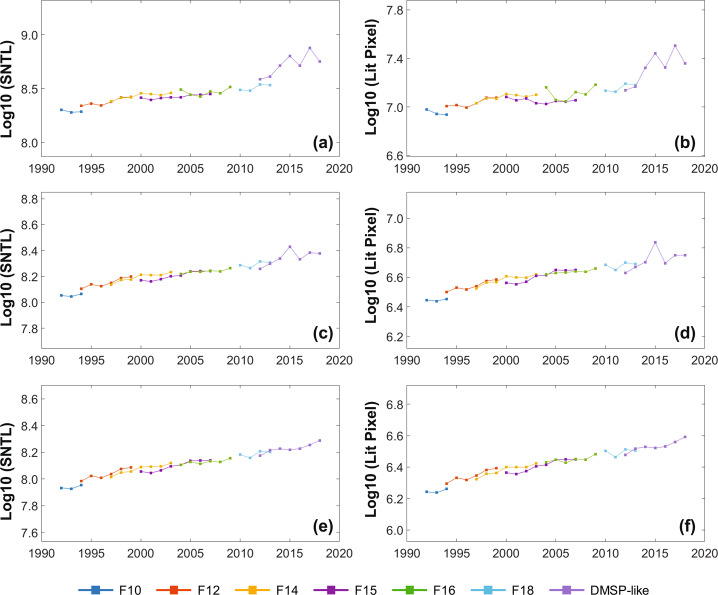
Temporal trends of harmonized NTL time series from 1992 to 2018 for the sum of NTL DN values and lit pixels using observations with DNs greater than 7 (**a**,**b**), 20 (**c**,**d**), and 30 (**e**,**f**), respectively.

The extended (2014–2018) global DMSP-like NTL time series data from the VIIRS data show a good agreement with historical observations from DMSP (1992–2013) (Fig. 5). Given that the minimum DN in our sigmoid model is 6.5, we calculated the SNTL and the number of lit pixels at the global scale using different thresholds of 7, 20, and 30. Overall, we observed a temporally more consistent trend of the integrated result from the DMSP (i.e., F10, F12, F14, F15, F16, and F18) and DMSP-like data, with the increase of accounting threshold. The VIIRS-derived results are more fluctuated during the period from 2015 to 2018 for pixels with DN values greater than 7 (Fig. 5a,b), and this fluctuation was notably mitigated for pixels with relatively high DNs. This suggests the converted DN values from VIIRS data are more reliable for pixels with DN values larger than 20 (Fig. 5c,d), and the derived temporal patterns of SNTL and lit pixels are more reliable for pixels with DN values above 30 (Fig. 5e,f). There are two possible reasons for the fluctuation of derived NTL time series data from VIIRS. First, the original observations of VIIRS NTL data fluctuate over years^24^. For example, the drop of SNTL and lit pixels from 2015 to 2016 in our result was also observed in the annual result of VIIRS data from the Payne Institute for Public Policy under the Colorado School of Mines^24^. Second, the simulated DMSP-like NTL data from VIIRS have a larger extent than the DMSP data with the improved sensitivity of sensors in VIIRS and the aggregation procedure using the kernel density method and point-spread function, especially in low luminance regions (Fig. S3). After excluding those low luminance regions using the threshold of 7, the derived NTL result from VIIRS is closer with the DMSP data. When the threshold increases to 20 and 30, most blooming effects around the city and villages are eliminated, resulting in a more continuous time series data of the SNTL and the number of lit pixels (Fig. 5). Similar patterns can also be found in the urban domain (Fig. S4). The generated extended DMSP-like NTL time series data can support a variety of studies such as dynamics of urban extents, electricity consumption, carbon emissions, and light pollution. It is worth to note that users should be cautious when applying the data in rural areas due to the uncertainty in low luminance regions.

The intercalibrated DMSP NTL time series (1992–2013) data is the foundation to implement the extension of NTL data using the new generation NTL observations from VIIRS. We generated a temporally consistent DMSP NTL time series dataset from 1992 to 2013 using a stepwise calibration approach^26^. The proposed approach outperforms other approaches in terms of the temporal consistency. The intercalibrated DMSP NTL time series (1992–2013) data have been validated using physical indicators of the sum of NTL and the number of lit pixels at the global and country scales. Also, the data show a good agreement with the temporal trend of socioeconomic activities (e.g., gross domestic product and electricity consumption).

### Spatial patterns of generated NTL time series

**Fig. 6:**
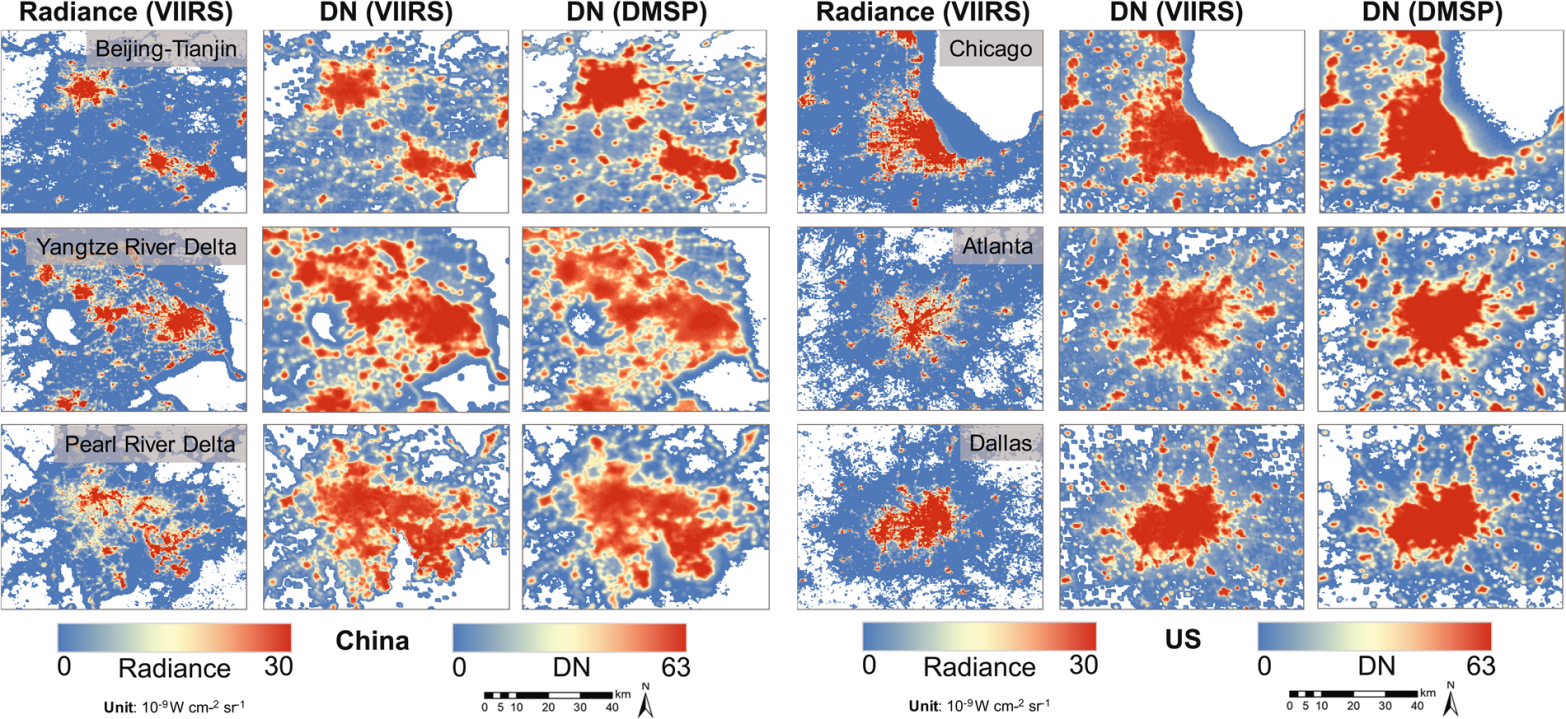
Spatial patterns of the raw radiance, DMSP-like DNs from VIIRS, and DNs from the inter-calibrated DMSP in example metropolitan areas in China and US in 2013.

**Fig. 7:**
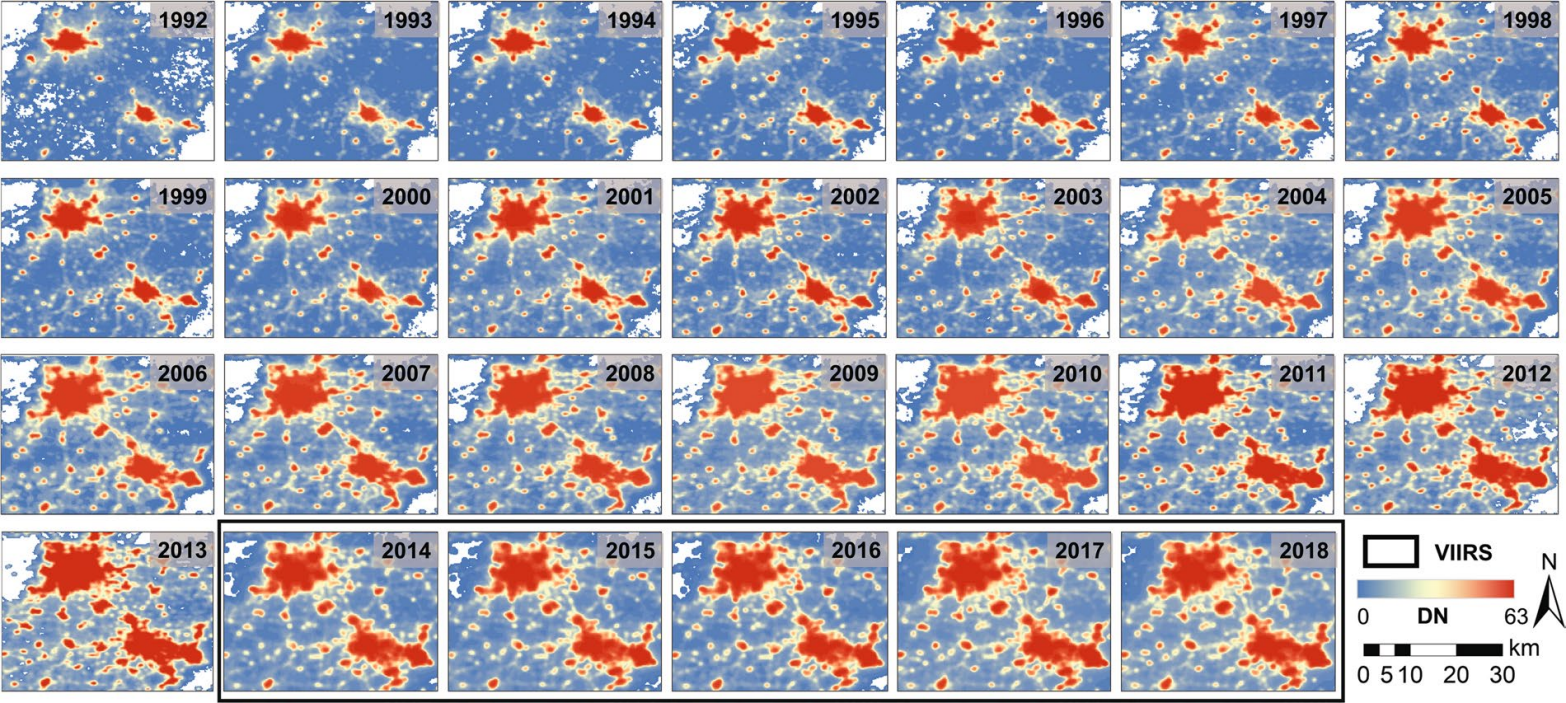
Spatial patterns of NTL time series in the Beijing-Tianjin metropolitan region in China. These images include the inter-calibrated DMSP data from F10 (1992–1994), F12 (1995–1996), F14 (1997–2003), F16 (2004–2009), F18 (2010–2013) and DMSP-like data from VIIRS (2014–2018).

The derived DMSP-like NTL data from VIIRS show similar spatial patterns as the original DMSP data (Fig. 6). Compared to the raw VIIRS radiance data, the spatial pattern of the DMSP-like data agrees better with the original DMSP data. In general, over past 27 years (1992–2018), the extended NTL time series exhibits a temporally consistent trend of both the increasing NTL and the continuous spatial expansion for high luminance pixels from the urban cores to fringe areas (Fig. 7).

## Usage Notes

The 27-year records of NTL time series data are of great potential for monitoring human activities across local, regional to global scales. The inter-calibrated DMSP NTL data (1992–2013) using the stepwise calibration approach shows a temporal consistent trend of NTL observations, and the DMSP-like NTL data derived from VIIRS (2014–2018) extended the observation span of historical DMSP NTL data. Although there are uncertainties in NTL pixels with DN values lower than 10 (Fig. 5), the influence of these uncertainties in low-DN areas on applications is limited because human activities with high luminance are always paid more attentions in studies using the stable DMSP NTL data. For example, the optimal threshold for delineating the urban boundary is generally larger than 30^17^. This dataset can be used to support studies on monitoring long-term dynamics of human activities, such as the urban expansion^17,32^, carbon emission^7,40^, electricity consumption^8,10,11^, and light pollutions^41,42^, from local to global scales.

## Supplementary information

Supplementary Figures

## Data Availability

The programs used to generate all the results were MATLAB (R2018b) and ArcGIS (10.4). Analysis scripts are available on request from Y.Z. (yuyuzhou@iastate.edu).
