# Supplementary material for: A harmonized global nighttime light dataset 1992–2018
Source: Sci Data. 2020 Jun 4;7:168. doi: 10.1038/s41597-020-0510-y (PMC7272434; doi:10.1038/s41597-020-0510-y)
Supplement: Supplementary file 1 — Supplementary Figures [file 41597_2020_510_MOESM1_ESM.docx]

# Title

*A harmonized global nighttime light data (1992-2018) from DMSP and VIIRS*

# Authors

Xuecao Li^1^, Yuyu Zhou^1*^, Min Zhao^1^, Xia Zhao^1^

**Affiliations**

1. Department of Geological and Atmospheric Sciences, Iowa State University, Ames, IA, 50011, USA

Corresponding author(s): Yuyu Zhou ([yuyuzhou@iastate.edu](mailto:yuyuzhou@iastate.edu))

**Supplementary Figures**

Fig. S1-S4

# Fig. S1


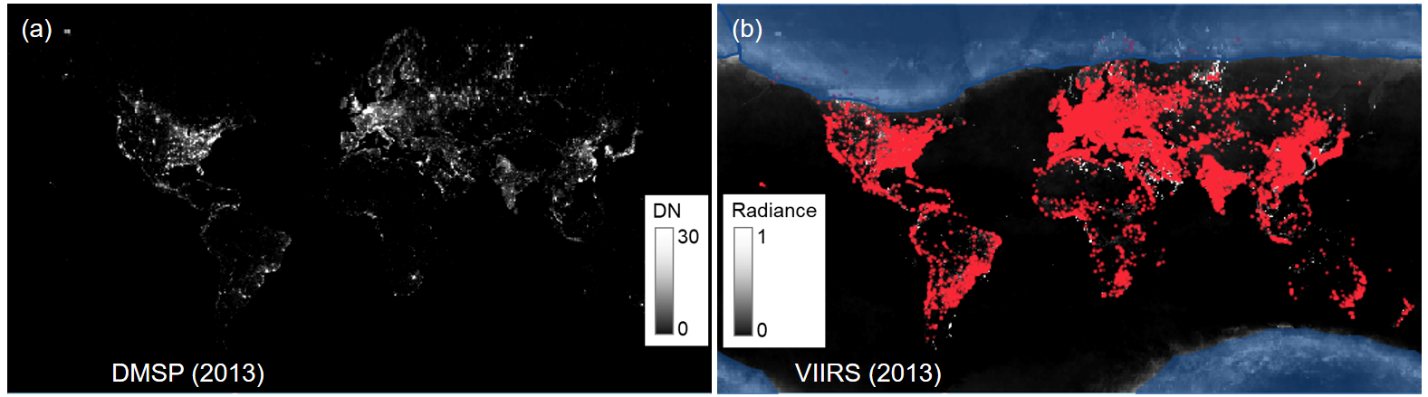


**Fig. S1**. Spatial distribution of DMSP data (a) and VIIRS data (b). The blue and red areas in (b) are zones affected by aurora and urban clusters, respectively.

# Fig. S2


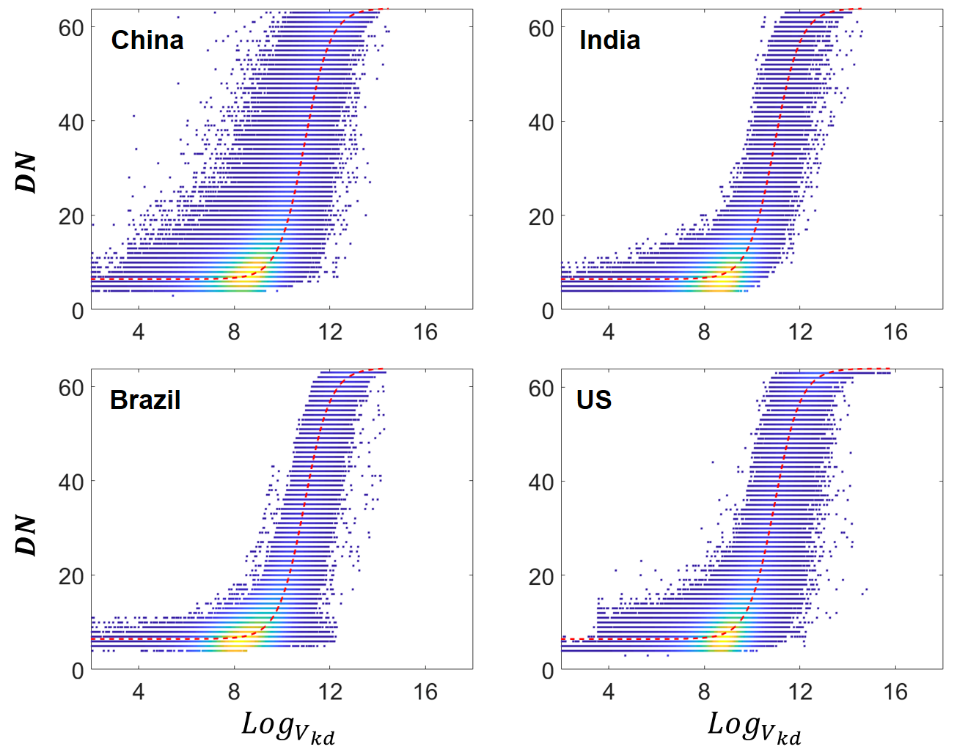


**Fig. S2**. Performance of the proposed sigmoid function in example countries.

# Fig. S3


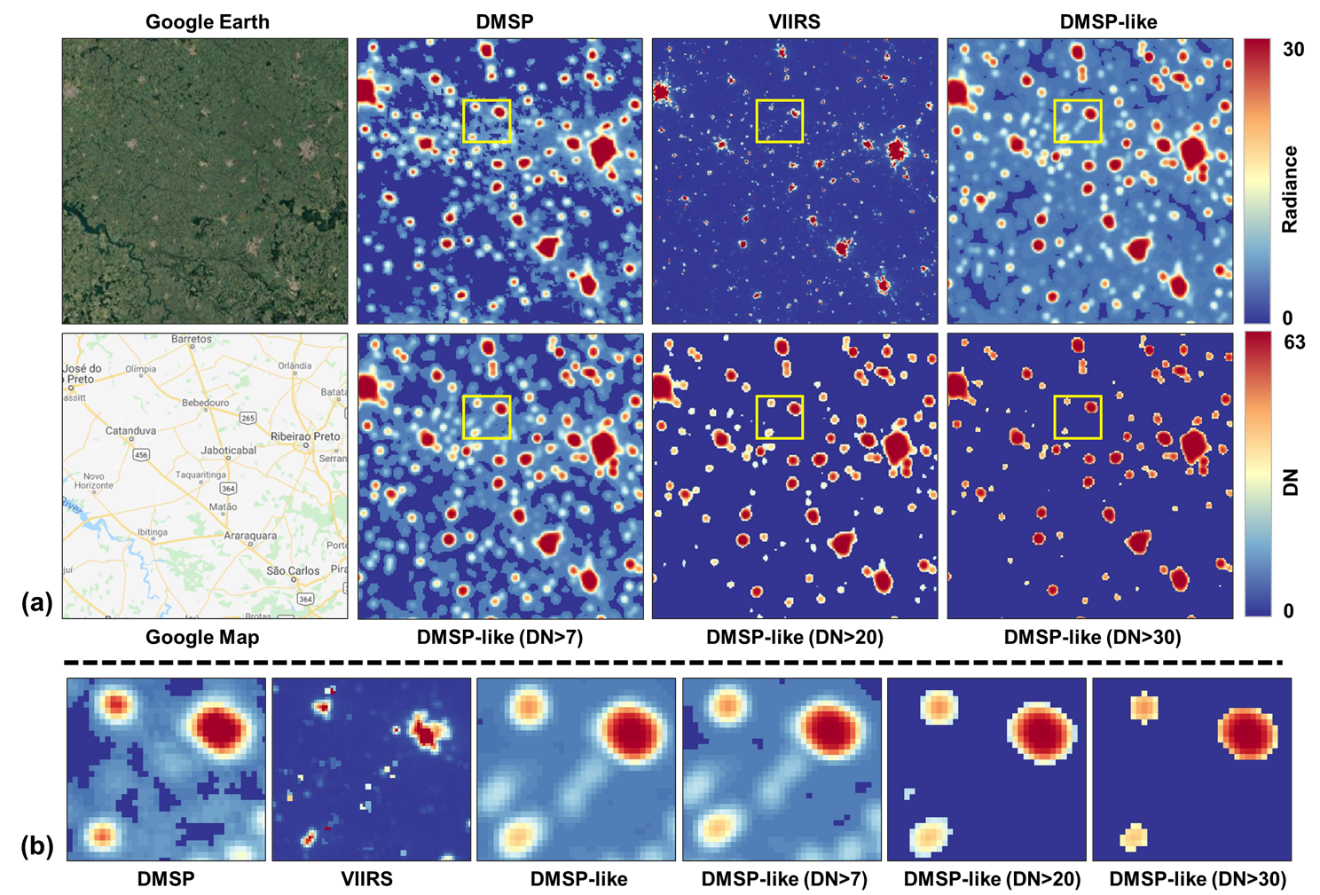


**Fig. S3**. Comparison between the simulated DMSP-like data from VIIRS and DMSP data in rural areas in Brazil, South America in 2013. The extent of subplots in (a) is 200km × 200km. Subplots in (b) are enlarged from the yellow frame in (a).

# Fig. S4


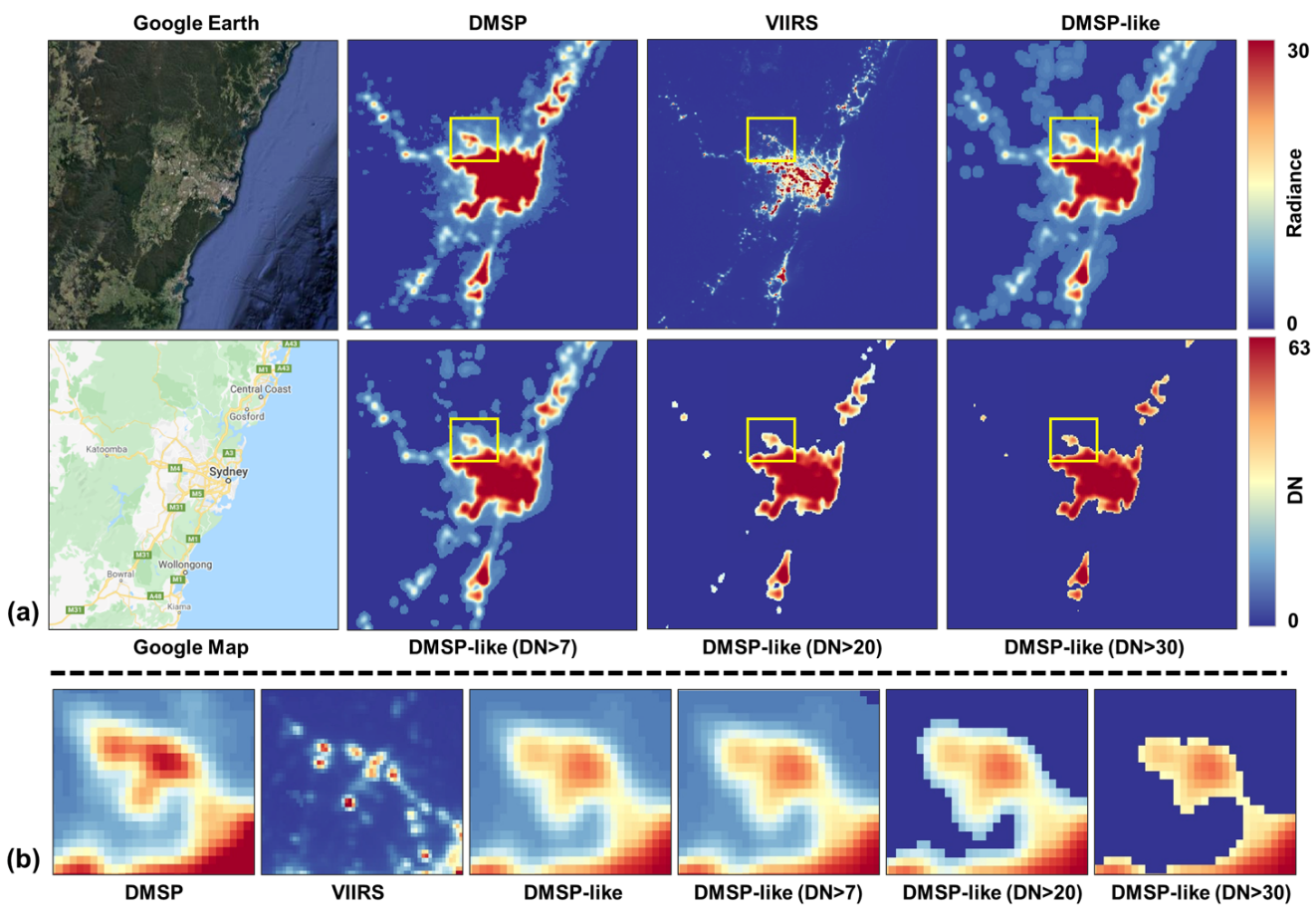


**Fig. S4.** Comparison between the simulated DMSP-like data from VIIRS and DMSP data in urban domain in Sydney, Australia in 2013. The extent of subplots in (a) is 200km × 200km. Subplots in (b) are enlarged from the yellow frame in (a).
